# Long-Term Clinical Outcomes of Thoracic Endovascular Aortic Repair for Arch Aneurysms with the Najuta Thoracic Stent-Graft System

**DOI:** 10.3400/avd.oa.20-00102

**Published:** 2020-12-25

**Authors:** Hiroshi Sato, Joji Fukada, Yukihiko Tamiya, Takuma Mikami, Tsuyoshi Sibata, Ryo Harada, Syuichi Naraoka, Takeshi Kamada, Nobuyoshi Kawaharada, Yoshihiko Kurimoto

**Affiliations:** 1 Department of Cardiovascular Surgery, Otaru General Hospital, Otaru, Hokkaido, Japan; 2 Department of Cardiovascular Surgery, Sapporo Medical University School of Medicine, Sapporo, Hokkaido, Japan; 3 Department of Cardiovascular Surgery, Teine Keijinkai Hospital, Sapporo, Hokkaido, Japan

**Keywords:** thoracic endovascular aortic repair (TEVAR), arch aneurysm, fenestrated stent graft

## Abstract

**Objective**: The treatment for arch aneurysms by endovascular repair is often difficult. This study evaluated the long-term outcomes of thoracic endovascular aortic repair for aortic arch aneurysms treated with the Najuta stent-graft system.

**Materials and Methods**: From January 2009 to December 2019, 37 patients underwent treatment for aortic aneurysms with the Najuta stent graft system at two institutes, including our hospital. We retrospectively analyzed the short- and long-term clinical outcomes.

**Results**: Of all 37 cases, the technical success rate was 97.3% (36 of 37). The mean proximal neck length was 20.1±5.3 mm. The postoperative results revealed 10 patients with type Ia endoleaks (27.8%), 6 with stroke (16.7%), and one with paraplegia (2.8%). In the chronic phase, the overall survival rates and the rates of freedom from aorta-related events at 7 years were 71.3% and 50.7%, respectively. Between two groups divided based on the proximal neck diameter of 20 mm, the <20-mm group had significantly higher rates of aorta-related events in terms of freedom from aortic events (P=0.046).

**Conclusion**: The fenestrated stent graft can be a less invasive option for the treatment of high-risk patients with aortic aneurysms.

## Introduction

Recently, thoracic endovascular aortic repair (TEVAR) has been established as the gold standard surgery for descending aortic aneurysms because it has shown very effective long-term outcomes.^1–3)^ However, an aortic arch aneurysm is often difficult to treat with TEVAR because of the arch curvature and the presence of supra-aortic vessels, so the first-line surgical treatment for arch aneurysms has been graft replacement, until now.^4)^ Although TEVAR is alternatively selected for high-risk patients in whom it is impossible to perform graft replacement, most of them are considered as very difficult cases for endograft repair because of the need for zone 0 or 1 proximal stent graft landing.^5)^ Several surgical techniques for arch aneurysms that require zone 0–1 landing have been reported, one of which is the Najuta thoracic stent-graft system. This fenestrated stent graft is a semicustom device made using preoperative three-dimensional computed tomography (CT) and can be utilized by deploying on the arch aorta without reconstruction for supra-aortic vessels because of the fenestration on the device.^6,7)^ Although this device allows for a less invasive and simple surgical procedure, device-specific disadvantages, such as low flexibility and low radial force, have been reported.^8)^ Its long-term clinical results are unclear and not well reported. The present study evaluated the clinical outcomes of TEVAR using the Najuta stent-graft system for aortic arch aneurysms that require zone 0–1 landing. This study has been approved by IRB of Otaru General Hospital, with approval number 02-003.

## Materials and Methods

### Patients

Patients who underwent TEVAR using the Najuta stent-graft system for arch aneurysms at two institutions, Otaru General Hospital and Sapporo Medical University School of Medicine, between January 2009 and December 2019 were included in the present study. Before the procedure, we obtained written informed consent from all patients. The proximal neck length was defined as the direct length from the origin of the preserving vessel to the proximal edge of the aneurysm, which was the site along the aortic wall.

### Surgical procedures

The details of the Najuta stent graft and procedure have been reported previously by Yokoi et al.^9)^ The common femoral artery was used as the access artery in all cases. The stent graft was inserted using a tug-of-wire method to avoid excessive arterial injury. The device was deployed under fluoroscopic guidance without additional circulatory support. Postdeployment touch-up ballooning was performed as required. The left subclavian artery (LSCA) was revascularized in selected patients, such as those at high risk of spinal cord ischemia.

### Definition and end points

The significant change of the aneurysmal diameter was defined as >5 mm from the first postoperative CT. The end points of this study included technical success, complications, overall survival rates, and rates of freedom from aorta-related events, including aneurysmal enlargement, stent-graft migration, rupture, and retrograde type A dissection (RTAD).

### Statistical analysis

The overall survival rates and the rates of freedom from aorta-related events were assessed using the Kaplan–Meier method. Furthermore, the rates of aorta-related events were compared using the log-rank test between two groups that were divided based on the proximal neck length of 20 mm. The logistic regression model was used to identify predominant risk factors of type Ia endoleak and stroke. The best model was selected by the backward step-down selection using the Akaike Information Criteria. All data analyses were performed using the statistical program R, version 3.2.1 (R Foundation for Statistical Computing; http://www.r-project.org/).

## Results

### Patients’ characteristics, aneurysmal configuration, and operative outcomes

Of all 37 patients, 29 were males (78.4%), and the mean age was 77.3±7.1 years. The mean follow-up period was 2.9±2.9 years. All the cases were considered as high risk for open surgical graft replacement. The patients’ characteristics are shown in **Table 1**. Regarding aneurysm configurations, the number of fusiform aneurysms was 20 (54.1%), that of saccular aneurysms was 13 (35.1%), that of true aneurysms was 33 (89.2%), and that of dissection was 4 (10.8%). The mean maximum aneurysmal diameter was 57.1±14.4 mm. The mean proximal neck length was 20.1±5.3 mm. The technical success rate was 97.3% (36 of 37; in one case, a guide wire got stuck in the main stent-graft device). The proximal landing zone of the stent graft was located at zone 0 in 31 patients (86.1%), zone 1 in 5 patients (13.9%), and zone 2 and 3–4 in 0 patients (0%). The fenestrated proximal neck vessel was the brachiocephalic artery (BCA) in 33 patients (91.7%), the left common carotid artery (LCCA) in 35 patients (97.2%), the LSCA in 8 patients (22.2%), and LSCA reconstruction in 16 patients (44.4%) (**Table 2**).

**Table 1:** Table 1 Patients’ characteristics

Variable	
Age (years)	77.3±7.1
Sex (male)	29 (78.4)
Medical history	
Hypertension	28 (77.8)
Dyslipidemia	6 (16.2)
Diabetes mellitus	11 (29.7)
COPD	7 (18.9)
Coronary artery disease	10 (27.0)
Cerebrovascular disease	5 (33.3)
CKD	4 (10.8)
Arrythmia	4 (10.8)
Previous cardiovascular surgery	14 (37.8)
History of cancer	8 (21.6)
EuroSCORE II	18.6±8.8
Etiology	
Degenerative aneurysm	
Fusiform	20 (54.1)
Saccular	13 (35.1)
Dissection	4 (10.8)
Aneurysm diameter (mm)	57.1±14.4
Aneurysm length (mm)	50.7±16.1
Proximal neck diameter (mm)	34.7±3.9
Distal neck diameter (mm)	29.5±3.4
Proximal neck length (mm)	20.1±5.3

The data are presented as the mean±standard deviation (SD) or n (%). COPD: chronic obstructive pulmonary disease; CKD: chronic kidney disease; EuroSCORE: European system for cardiac operative risk evaluation

**Table 2:** Table 2 Surgical procedure and results

Variable	
Technical success	36 (97.3)
Zone classification	
0	31 (86.1)
1	5 (13.9)
2	0 (0)
3–4	0 (0)
Proximal fenestrated vessel	
BCA	33 (91.7)
LCCA	35 (97.2)
LSCA	8 (22.2)
LSCA reconstruction	16 (44.4)
Short term	
Stroke	6 (16.7)
Paraplegia	1 (2.8)
Type Ia endoleak	10 (27.8)
SINE	0 (0)
RTAD	0 (0)
Hospital stay (days)	19.5±15.7
In-hospital mortality	0 (0)
Long-term	
Change of aneurysm size	
Enlargement	10 (27.8)
No change	15 (41.7)
Shrinkage	11 (30.6)
Secondary intervention	3 (8.3)
Aortic event	9 (25.0)
Mortality	4 (11.1)
Follow-up period (years)	2.9±2.9

The data are presented as the mean±SD or n (%). BCA: brachiocephalic artery; LCCA: left common carotid artery; LSCA: left subclavian artery; SINE: stent-graft-induced new entry; RTAD: retrograde type A aortic dissection

### Short- and long-term outcomes

**Figure 1:**
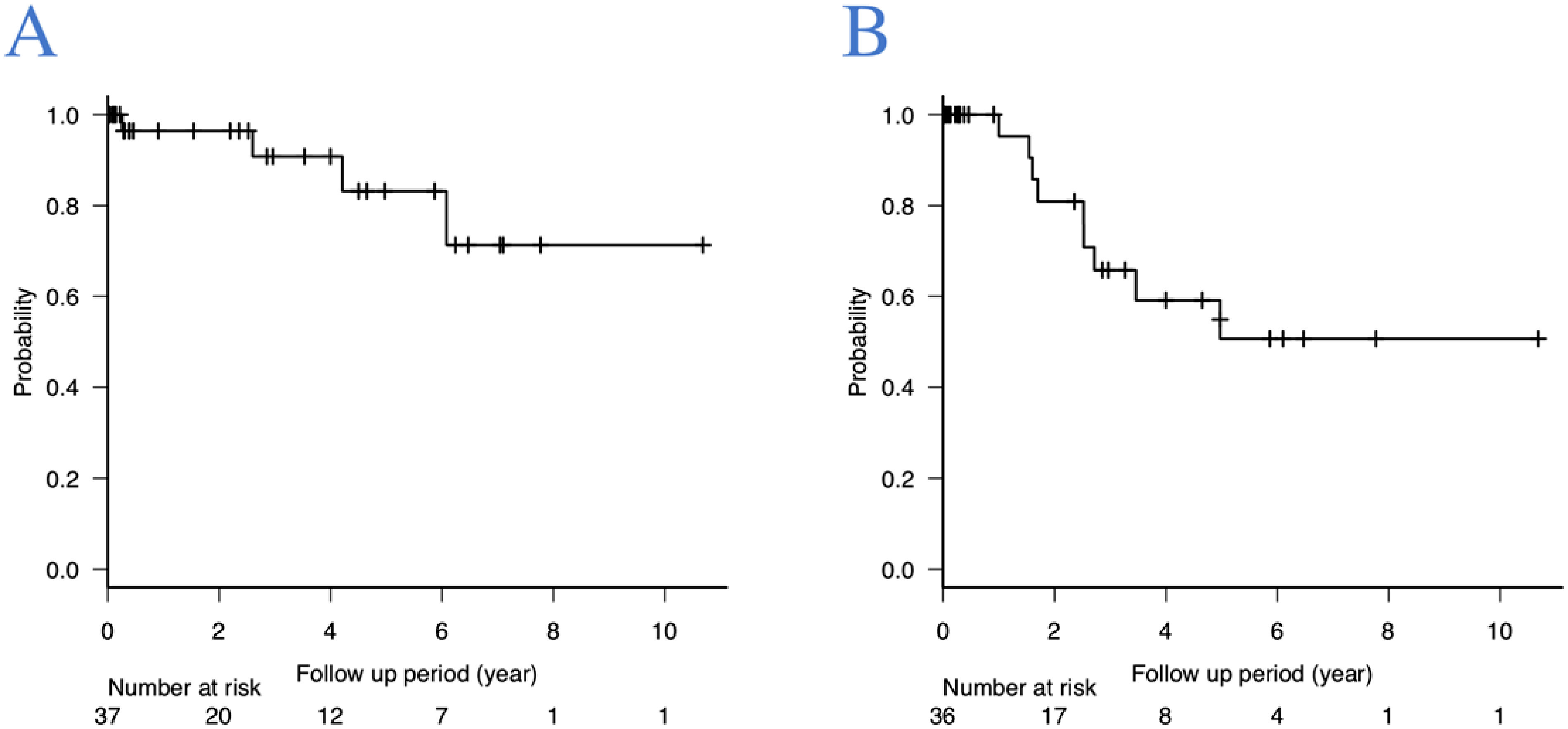
Fig. 1 Kaplan–Meier curves showing the overall survival rates and the rates of freedom from aorta-related events after TEVAR using the Najuta fenestrated stent-graft system. (**A**) Overall survival rates at 1, 3, 5, and 7 years were 96.4%, 90.8%, 83.2%, and 71.3%, respectively. (**B**) Rates of freedom from aorta-related events at 1, 3, 5, and 7 years were 90.5%, 65.8%, 50.7%, and 50.7%, respectively.

**Figure 2:**
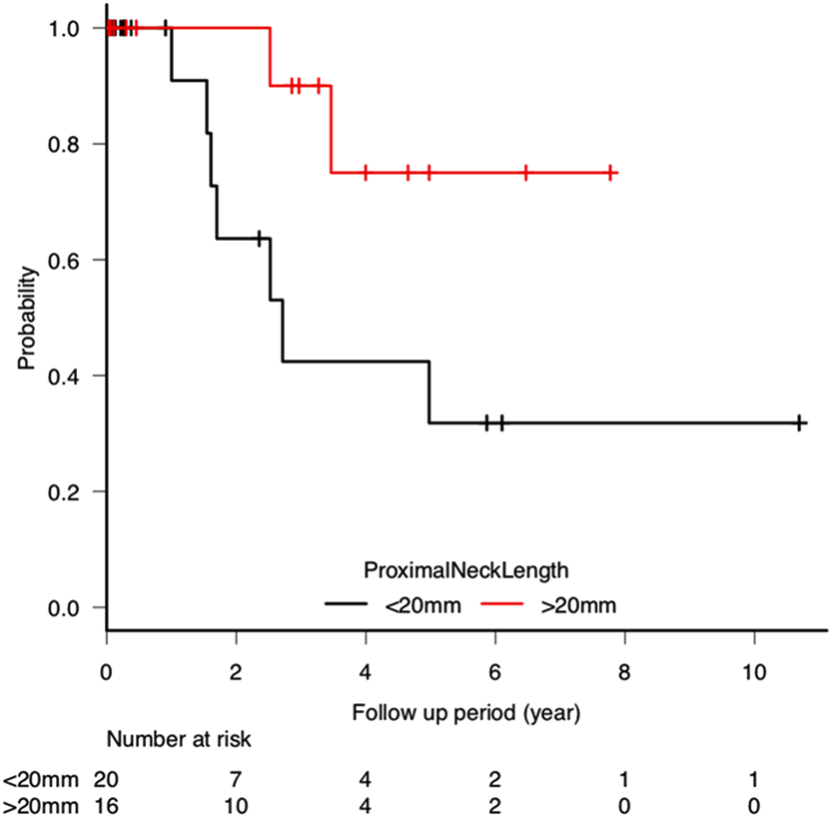
Fig. 2 Kaplan–Meier curve showing the rates of freedom from aorta-related events in the two groups divided based on the proximal neck length of 20 mm. The <20-mm group shows significantly lower rates of freedom from aorta-related events (log-rank test: P=0.046).

In the postoperative results, six patients had strokes (16.7%) and one had paraplegia (2.8%). There was neither stent-induced new entry nor RTAD. Type I a endoleaks were recognized in 10 patients (27.8%). The in-hospital mortality was 0%. In the chronic phase, the aneurysmal size shrank in 11 patients (30.6%), there was no change in 15 patients (41.7%), and it was enlarged in 10 patients (27.8%). Four patients died during the follow-up owing to malignant tumors (2), RTAD (1), and unknown cause (1) (**Table 2**). Three patients underwent reinterventions, which consisted of coil embolization (two patients) and re-TEVAR for stent graft migration (one patient). Kaplan–Meier curves show that the overall survival rates and the rates of freedom from aorta-related events at 1, 3, 5, and 7 years were 96.4%, 90.8%, 83.2%, and 71.3% and 90.5%, 65.8%, 50.7%, and 50.7%, respectively (**Fig. 1**). Between the two groups divided based on the proximal neck diameter of 20 mm, the <20-mm group had significantly higher rates of aorta-related events in terms of freedom from aortic events (P=0.046) (**Fig. 2**). The results of the univariate and multivariate logistic regression analyses revealed that the proximal neck length was the risk factor for type Ia endoleak, and previous stroke and diabetes mellitus were detected as risk factors for stroke (**Table 3**).

TEVAR: thoracic endovascular aortic repair

**Table 3:** Table 3 Risk factors for type Ia endoleak and stroke identified by univariate and multivariate logistic regression analyses

Dependent variable	Independent variables	Univariate			Multivariate		
		OR (95%CI)		P-value	OR (95%CI)		P-value
Type Ia endoleak	Age (years)	0.985	(0.89–1.09)	0.763			
	Sex (male)	0.556	(0.11–2.94)	0.49			
	Arrythmia	0.852	(0.08–9.3)	0.895			
	Chronic kidney disease	3	(0.36–24.9)	0.309			
	Chronic obstructive pulmonary disease	0.37	(0.04–3.54)	0.389			
	Dyslipidemia	3.29	(0.54–20.1)	0.198			
	Diabetes mellitus	2.22	(0.47–10.6)	0.316			
	Hypertension	2.1	(0.5–5.6)	0.994			
	Coronary artery disease	0.25	(0.04–2.32)	0.223			
	Previous stroke	2.25	(0.51–10)	0.287			
	Previous cardiovascular surgery	4.07	(0.88–18.9)	0.0727			
	Fusiform	1.29	(0.29–5.66)	0.031			
	Aneurysm diameter (mm)	0.996	(0.95–1.05)	0.866			
	Aneurysm length (mm)	1	(0.95–1.05)	0.977			
	Proximal neck length (mm)	0.828	(0.68–1.01)	0.021	0.83	(0.68–0.95)	0.039
	Proximal neck diameter (mm)	1.14	(0.93–1.41)	0.039			
	LSCA reconstruction	1.36	(0.32–5.9)	0.678			
	Proximal fenestrated vessel (BCA, LCCA, LSCA)	0.7	(0.1–1.3)	0.98			
Stroke	Age (years)	0.958	(0.85–1.08)	0.47			
	Sex (male)	1.52	(0.15–15.3)	0.72			
	Arrythmia	1.8	(0.15–21)	0.64			
	Chronic kidney disease	1.8	(0.15–21)	0.64			
	Chronic obstructive pulmonary disease	0.8	(0.08–8.19)	0.85			
	Dyslipidemia	1	(0.1–10.5)	1.00			
	Diabetes mellitus	25	(2.38–263)	0.007	2.5	(1.38–5.1)	0.007
	Hypertension	1.59	(0.16–16)	0.69			
	Coronary artery disease	1.64	(0.25–10.9)	0.61			
	Previous stroke	18	(3.4–11.8)	0.003	2.4	(1.2–4.9)	0.015
	Previous cardiovascular surgery	2	(0.34–11.8)	0.44			
	Fusiform	0.765	(0.13–4.43)	0.77			
	Aneurysm diameter (mm)	1.01	(0.95–1.07)	0.80			
	Aneurysm length (mm)	1	(0.94–1.06)	1.00			
	Proximal neck length (mm)	1.05	(0.9–1.23)	0.55			
	Proximal neck diameter (mm)	0.823	(0.62–1.1)	0.19			
	LSCA reconstruction	3	(0.47–19)	0.24			
	Proximal fenestrated vessel (BCA, LCCA, LSCA)	0.8	(0.1–1.2)	0.99			
	Blood loss (ml)	0.72	(0.72–1.6)	0.56			
	Packed red blood cells (ml)	0.82	(0.65–1.8)	0.66			

OR: odds ratio; CI: confidence interval; BCA: brachiocephalic artery; LCCA: left common carotid artery; LSCA: left subclavian artery

## Discussion

Graft replacement for arch aneurysms with cardiopulmonary bypass can be difficult to perform in patients with poor health conditions, as it is a highly invasive procedure. Although endovascular therapy is selected for these patients, TEVAR, which has shown effective results in the treatment of descending aortic aneurysms, is not always good for the treatment of arch aneurysms. This is because of the difficulty of device fitting in the arch curvature and the insufficient proximal landing length owing to the presence of supra-aortic branches. These difficulties can be the causes of the incidence of endoleaks and later aneurysmal enlargement.

Surgical procedures, such as the chimney technique and debranched technique, have been reported to preserve the supra-aortic branch blood flow with TEVAR from zone 0 to 1. Either of these techniques can treat arch aneurysms without graft replacement. However, in the chimney technique, type Ia endoleak may occur because of the gap between the chimney graft and the main graft. Thus, the rate of type Ia endoleaks has been reported from 19.7% to 40.0%.^10,11)^ In contrast, in the debranched technique, a sufficient proximal landing length can be achieved by branch bypass, but the surgical procedure for BCA or LCCA can cause cerebral infarction, which has been reported in 11.4%–26.9% cases.^4,12,13)^ Thus, these techniques can be surgical options for arch aneurysms, but not first-line treatments.

The precurved Najuta fenestrated endograft was developed to solve these clinical issues in a completely endovascular manner.^9)^ The incidence of cerebral infarction was reported to be 0%–5.4%, which is lower than, and superior to, the other abovementioned techniques.^5,8,14)^ However, there are structural device disadvantages, such as the lack of conformability of stents 25 mm in length and low radial force by the absence of the stent at the fenestrated lesion. Even if the proximal landing is from the ascending aorta, the proximal sealing can often be inadequate. For this reason, the incidence of type Ia endoleaks was higher than that of other devices, which has been reported to be 4.2%–32.4%.^5,9,14)^ In the study of Kurimoto et al., which summarized the results of treatment using the Najuta stent graft for 37 patients with arch aneurysms, the incidence of type Ia endoleaks was 32.4%, and the rates of freedom from aorta-related events at 2 and 5 years were 88.5% and 56.5%, respectively.^14)^ In the present study, these results are almost the same: the incidence of type Ia endoleaks was 27.8%, and the rates of freedom from aorta-related events at 2 and 5 years were 81.0% and 50.7%, respectively. Furthermore, the group with the proximal neck length <20 mm showed a higher rate of aortic events in the chronic phase. Further, a short proximal neck length was identified as an independent risk factor of type Ia endoleaks (**Table 3**). This is because a length <20 mm is inadequate as proximal sealing, and it has indicated the clinical limitation of treatment with the Najuta stent graft. According to the suggestion from instructions for use, it seems better to avoid using it for patients with proximal neck length <20 mm.

In summary, the Najuta stent graft can be considered for use in patients at high risk for total arch replacement and surgical procedure for cerebral vessels by the debranched or chimney techniques, and with a proximal neck length >20 mm.

Although the clinical outcome of the present study is not better than other general TEVAR results, we have considered it almost acceptable as a less invasive treatment option for high-risk patients who are unable to undergo graft replacement. However, the surgical procedure for total arch replacement has been established and has shown improved clinical outcomes recently. Previous studies have reported a stroke rate of 2.4%–8.0%, an in-hospital mortality rate of 4.9%–5.4%, survival at 5 years of 70%–90.1%, and the rate of aorta-related events as 0%.^12,15)^ These good results may suggest expanding the indication of total arch replacement to high-risk patients. It will be necessary to select the surgical strategy in full consideration of these results.

There were several limitations in this retrospective, two-center study. Multivariate analysis was not performed owing to the small number of cases. Thus, statistical reliability may be insufficient. Furthermore, because the application of TEVAR was often judged depending on each operator, whether graft replacement was really impossible or not remains unknown. Improvements in the quality of analyzed data and prospective data accumulation for elimination of data bias are required.

## Conclusion

We have summarized the long-term clinical outcomes of TEVAR using the Najuta stent-graft system. Although our results were not better because of the high incidence of type Ia endoleaks and aortic events in the chronic phase, we have considered TEVAR acceptable as a less invasive treatment option for high-risk patients who are unable to undergo graft replacement.
